# Resistive Chemosensors for the Detection of CO Based on Conducting Polymers and Carbon Nanocomposites: A Review

**DOI:** 10.3390/molecules27030821

**Published:** 2022-01-26

**Authors:** Mihaela Savin, Carmen-Marinela Mihailescu, Carmen Moldovan, Alexandru Grigoroiu, Ion Ion, Alina Catrinel Ion

**Affiliations:** 1 Department of Microsystems in Biomedical and Enviromental Applications, National Institute for Research and Development in Microtechnologies, Erou Iancu Nicolae Street 126A, 077190 Bucharest, Romania; mihaela.savin@imt.ro (M.S.); carmen.moldovan@imt.ro (C.M.); alexandru.grigoroiu@imt.ro (A.G.); ion.ion@upb.ro (I.I.); 2 Department of Applied Chemistry and Materials Science, University Politehnica of Bucharest, 1-7 Polizu, 011061 Bucharest, Romania

**Keywords:** chemosensor, carbon monoxide, nanocomposite carbon material, sensing mechanism, electrodeposition, conducting polymers

## Abstract

Nanocomposite materials have seen increased adoption in a wide range of applications, with toxic gas detection, such as carbon monoxide (CO), being of particular interest for this review. Such sensors are usually characterized by the presence of CO absorption sites in their structures, with the Langmuir reaction model offering a good description of the reaction mechanism involved in capturing the gas. Among the reviewed sensors, those that combined polymers with carbonaceous materials showed improvements in their analytical parameters such as increased sensitivities, wider dynamic ranges, and faster response times. Moreover, it was observed that the CO reaction mechanism can differ when measured in mixtures with other gases as opposed to when it is detected in isolation, which leads to lower sensitivities to the target gas. To better understand such changes, we offer a complete description of carbon nanostructure-based chemosensors for the detection of CO from the sensing mechanism of each material to the water solution strategies for the composite nanomaterials and the choice of morphology for enhancing a layers’ conductivity. Then, a series of state-of-the-art resistive chemosensors that make use of nanocomposite materials is analyzed, with performance being assessed based on their detection range and sensitivity.

## 1. Introduction

Declining air quality has a great effect on modern lifestyles, with an estimate of 4.2 million deaths being attributed to ambient air pollution related diseases. The main contributors to the onset of diseases such as stroke, lung cancer, or acute respiratory diseases include carbon monoxide (CO), nitrogen oxides (NO_x_), sulfur oxides (SO_x_), and ammonia (NH_3_). The selective detection and monitoring of air, both from the ambient and indoor locations, could lead to great strides in limiting the exposure of people to toxic quantities of such gases, ensuring a favorable environment for development and growth. Of particular interest is CO, a colorless, odorless, and tasteless gas, resulting mainly from the incomplete combustion of fossil fuels and thus largely spread in urban environments or regions with a high traffic density. Both the molecular structure and chemical activity of carbon monoxide assure that irreversible bounds are formed when it interacts with hemoglobin, blocking the reaction sites that were originally meant for CO_2_. The resulting carboxyhemoglobin hinders gas exchange between carbon dioxide and oxygen, which can lead to death by functional asphyxia. Meanwhile, large concentrations, 0.1% for 1 h, of CO are lethal; the more problematic aspects of CO are its effects on the human health and psyche when under continuous exposure at low concentrations. Such cases routinely affect the central nervous system, reducing visual and physical capacity, lowering coordination, and introducing lapses in concentration. Thus, the World Health Organization (WHO) has prescribed exposures to CO of no more than 8 h at 9 ppm and 1 h at 26 ppm [1].

According to the IUPAC, chemical sensors can be classified according to their transduction mechanism [2] into three main classes: (i) systems that detect changes in the electrical and electrochemical properties of an analyte, (ii) systems that monitor changes in their physical properties, and (iii) sensors that measure the optical absorption of chemical analytes [3].

A subtype of the first class of chemical sensors, chemoresistive systems, when exposed to gas, change their electrical resistance due to the sensitive film deposited on their surface. An attractive feature of such sensors is the possibility of creating highly selective chemical films, which are tailored toward the detection of a specific gas, without a need of sweeping changes in the structure of the transducer or readout device. Prior research has identified composite materials, a solution of metal oxides, carbon nanostructures, and conducting polymers, as being effective films in the detection of toxic gases and in particular CO. The detection of gases by chemoresistive sensors has received great attention because of their advantages over the other sensors (electrochemical, optical): low cost, long lifetime, high sensitivity, fast response time, and small sizes [4].

Carbon nanomaterials, such as single-walled carbon nanotubes (SWCNTs), pristine carbon nanomaterials, multi-walled carbon nanotubes (MWCNTs), and graphene, have been extensively used as the active layers for the development of chemoresistive sensors [5,6,7]. This shift in material usage has been motivated by Kong et al., who observed a change in the conductivity of carbon nanotube (CNT) functionalized materials on gas absorption, leading to their use in a range of gas sensors [8,9]. Thus, polarity of the change in conductivity of carbonaceous-modified chemosensors becomes a function of the type of gas being analyzed. Hence, in highly oxidizing gases, electrons acceptor groups, such as those present in NO_2_ and CO, lead to increases in the conductivity of p-semiconductor carbon nanotubes due to an increase in the number of holes [10,11]. Meanwhile, in reducing gases, the presence of electron donors, such as in NH_3_, leads to a decrease in conductivity due to the occupation of holes with electrons. Thus, research is being conducted on combining carbonaceous materials with conductive polymers to form nanocomposite materials (NCMs) with increased selectivity to target gas molecules. With most chemosensors having an interdigitated electrode (IDE) substrate, a controllable deposition method is required. One such technique is electrochemical deposition, due to its high degree of control on the end parameters of the polymer film, such as topography and thickness [12,13].

Thus, the focus of this review will be on resistive chemosensors for CO detection with a functional layer based on a combination of carbon-based NCMs and conductive polymers (CP). The performance of three conductive polymers is analyzed in the following sections: polyaniline (PANI), polypyrrole (PPy), and poly (3,4-ethylenedioxythiophene) (PEDOT), in combination with SWCNTs, MWCNTs, and graphene oxide (GO) or reduced graphene (rGO). Analysis was performed from the perspective of sensing principle and the formulation strategies.

## 2. Chemosensors: An Overview on CO Detection

A versatile subset of chemosensors, those based on a composite material sensing layer, are primarily composed of three elements: metal oxides for the selective reaction with a target gas, carbon nanostructures for increasing the working area, and CP for increased stability and easier delivery of the signal to the transducer. Metal oxides refer to those p or n-type semiconductor materials, such as ZnO, SnO_2_, or CuO, that have good chemical stability, high electron mobility and that allow for easy control of their morphological properties. Their primary function consists in the absorption and reduction of a gas on their surface, which is followed by the measurement of any resulting change in the electrical conductivity and resistance of the sensing layer. While such sensing layers are highly sensitive, large-scale implementation has been previously limited by their high operating temperatures (300–500 °C) [14,15,16].

NCMs consisting of combinations of metal oxides with carbonaceous materials such as rGO, MWCNT, SWCNT, or other CNTs-based structures were the primary solution to the concerns regarding high temperature operation. Such structures operate at room temperature without a loss in sensitivity, thus proving a superior alternative to sensors solely based on metal oxides. Further enhancements in performance have been observed on doping a CP using metal oxides while at the same time functionalizing the polymer by use of carbon nanostructures. The use of functionalized conducting polymers such as PANI, polythiophene (PTh), PPy, and PEDOT leads to larger sensing surface areas and an increase in conductivity of the sensing layer due to the presence of many suspended bands and defects at their surface. However, such sensors are sensitive to changes in humidity, with high operating temperatures being employed to reduce its interference for simple metal oxide sensors and chemical alterations of the sensing surface being employed for composite materials.

**Figure 1:**
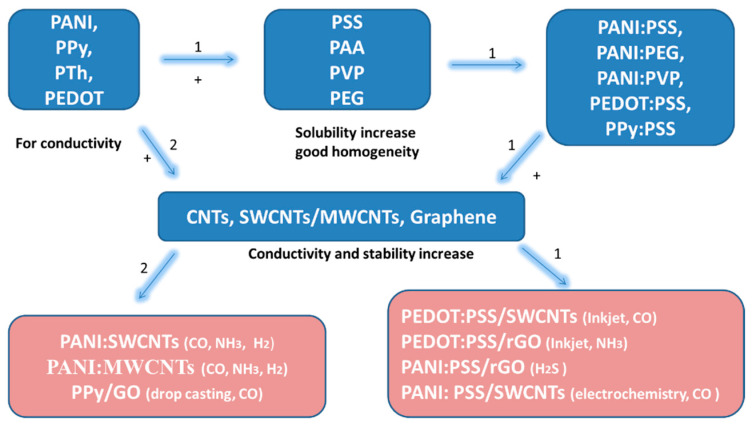
Effect of different reagents (polyelectrolytes) over the electrical proprieties of the conductive layers; abbreviations: polystyrene sulfonate (PSS), polyacrylic acid (PAA), polyvinylpyrrolidone (PVP), polyethylene glycol (PEG), polyaniline (PANI), poly(3,4-ethylenedioxythiophene)-PEDOT, reduced graphene oxide (r GO), single walled carbon nanotube (SWCNT), multi walled carbon nanotube (MWCNT), polypyrrole (PPy), graphene oxide (GO), carbon nanotubes (CNTs).

The use of conductive organic polymers has shown increased performance in the detection of gases, being highly synergistic with the functionalizing carbon nanostructures and preserving the individual properties of the constituting components. Polymers such as PANI, PTh, PEDOT, and PPy have shown superior electrical and mechanical properties when combined with CNTs or rGO, improving sensitivity by up to 100% [17,18,19]. However, to achieve those high-performance parameters, either a good adhesion of the metals to the CP or a high homogeneity of the CP solution needs to be assured. This is typically achieved by combining the organic CPs with hydrophilic polymers, with the solubility of PANI for example being increased by combinations with polystyrene sulfonate (PSS), polyacrylic acid (PAA), polyvinylpyrrolidone (PVP), or polyethylene glycol (PEG), leading to the formation of composite solutions such as PANI:PSS, PANI:PAA, PANI:PVP, and PANI:PEG, respectively. However, the homogeneity of the nanocomposite material dispersion can prove problematic due to CNTs having a poor solubility due to their hydrophobic nature. Thus, the uniformity of carbon structures, such as SWCNTs, within the CPs can be induced through methods such as SWCNTs functionalization, surfactants addiction, ultrasonication, or the association with other polymers, biomolecules, and organic acids. A diagram highlighting the effect of various reagents on the electrical proprieties with an increase gas sensitivity of such NCMs sensing layers can be seen in Figure 1.

**Figure 2:**
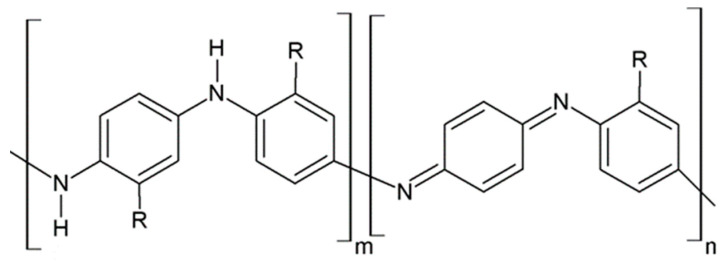
Oxidation of PANI: m = 1; n = 0, leucoemeraldine state, low conductivity; m = 0.5; n = 0.5, emeraldine state, high conductivity; m = 0, n = 1, pernigraniline state, low conductivity.

Regarding their chemical structures, all CPs have aromatic rings and display p electron conjugation, as shown in Figure 2. All three CPs have the capability to bind different NCMs for improved conductivity, stability, and selectivity. Table 1 presents the structures of all carbon-based NCMs employed in research for gas sensing.

Alternating single and double bonds for these CPs lead to a broadening of the p electron conjugation and thus to a decrease in the band energy and the stabilization of the molecule. Differences between the structures of the analyzed polymers are given by the presence of a nitrogen atom in the aromatic ring for PPy, whereas for PANI, a nitrogen atom is found outside the aromatic ring and a sulfur atom is found in the aromatic ring for PEDOT. The stabilization and electron insulation of the polymers is ensured by the electrons localized in the σ bonds in the CP chain, and due to the delocalization of the electrons within the p bonds, the CPs preserve conducting properties. However, high conductivity is assured by doping at the conjugated double bonds, which is a process analogous to the semiconductor doping process. For PPy and PEDOT, a redox reaction can be employed for doping, whereas for PANI, protonation is typically used. A common fabrication technique is the use of tethered oxygen or nitrogen atoms as they contain moieties that serve as effective tethering points for catalytically active additives to the CNTs composites. Fabrication of the sensing layer is achieved by electrodeposition, with conducting polymers being fixed on a conductive substrate with the help of an electrochemical cell. The morphology of the sensing surface is controlled through the tuning of the concentrations of the solvents, salts, and monomers in the electrodeposition process, while surface roughness is controlled by varying the deposition time or the charge–current ratio. The most common CPs employed in emerging CO sensing technologies are PANI, PTh, and PPy, which is due to their high electrical conduction, low cost, ease of fabrication, and flexibility in both use and structure. Depending on the electropolymerization method and the chosen parameters for the electrodeposition process, large changes in the conductivity and sensitivity to CO can be observed [20,21].

## 3. Sensing Principle

### 3.1. PANI Structure and Conductivity

PANI is an intrinsically conductive polymer that owes its electrical conductivity to the presence of a π-type electronic conjugation in its structure. One of the most studied polymers in the last 20 years, it can be employed for the detection of CO and other gas molecules such as NH_3_, H_2_S, and H_2_ having been integrated in both electronic and optical sensors [22,23,24]. While displaying a relatively low conductivity value (30–200 S·cm^−1^) when compared to other CPs such as doped polyacetylene or doped polyphenylene, PANI is preferable to those alternatives due to its high stability and multiple fabrication paths. Chemically, PANI consists of repeating units of benzene and quinoid rings. PANI can be employed in several of its redox forms, such as leucoemeraldine, emeraldine, and pernigraniline, with differences between the homologous forms being given by their oxidation state, which are expressed by using the value of m, as shown in Figure 2.

The conductive structures of PANI are predominantly composed of imine groups, although amine groups can also appear when the polymer is protonated in the presence of an acid or a dopant. This leads to the formation of polarons and bipolarons that are responsible for preserving the conductivity of PANI on substrates. Gas sensors based on PANI have shown high specificity for the detection of acidic and basic gases, such as NH_3_, H_2_S, and H_2_, for which they display a high sensitivity due to chemical reversibility of the acid–basic reaction being possible on the surface of the sensor. An overview of the performance of PANI-based sensors for the detection of CO can be seen in Table 2.

### 3.2. PANI Sensing Mechanism on CO Exposure

**Figure 3:**
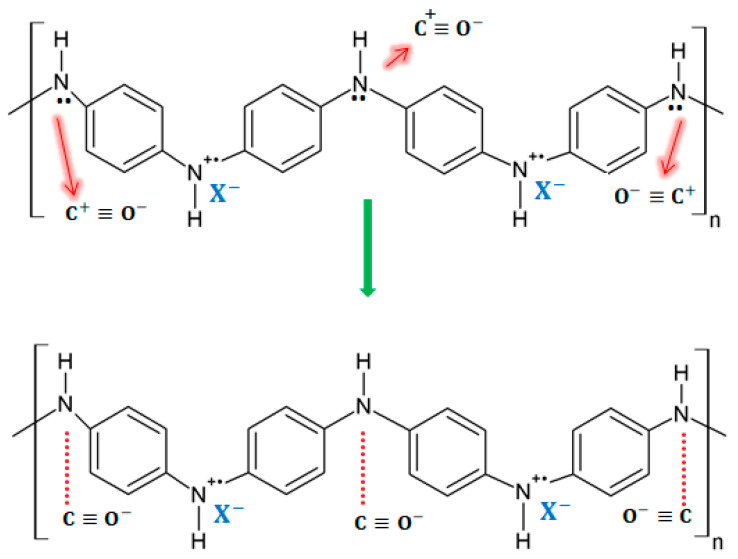
Sensing mechanisms for PANI in the presence of CO [31].

On exposure to CO, the response of a PANI-coated sensor consists of a decrease in the electrical resistance of the sensing layer due to the partial charge transfer between the amino nitrogen (–NH) structure in the polymer and the carbocation present in CO. Then, the transferred charge extends along the polymer chain, thus leading to an increase in the conductivity of the layer. A diagram of the sensing mechanism for the exposure of PANI to CO can be seen in Figure 3.

While most chemosensors based on PANI layers quantify the concentration of a target gas based on resistive measurements, forays have been made into other metrics such as current flow on CO exposure [23,24]. For such sensors, the current flow between the intercrystallite grain boundaries of PANI is linked to the gas concentration, with the polymer layer requiring deposition through vacuum-deposited nanocrystalline polyaniline for this functionality to be enabled. While such sensors show a rapid response at room temperature, the low contact area with CO molecules leads to saturation occurring rapidly, thus limiting the maximum measurable concentrations of CO to 150 ppm. The limit on saturation can be improved through the doping of the polymer, one example being maleic acid (MA)-doped PANI [27]. Then, the addition of carbon nanotubes (CNTs) to the sensing solution can lead to significant rises in sensitivity, due to an increase in the surface to volume ratio created by structures such as CNTs.

**Figure 4:**
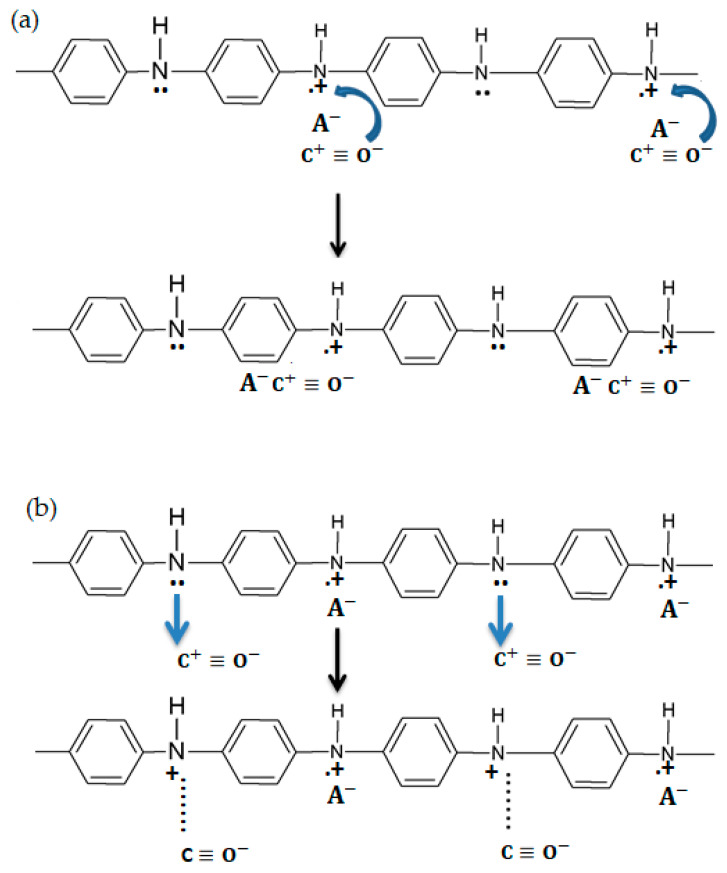
The proposed PANI-CO interaction mechanisms that cause the increase in electrical conductivity: (**a**) the active site is $\begin{array}{c} •+\\ \;\;-\mathrm{NH}- \end{array}$ or polaron; (**b**) the active site is $\begin{array}{c} ••\\ -\;N\;H- \end{array}$.

To investigate the conduction mechanisms of PANI blend with polyimide (PI), Watcharaphalakorn et al. employed in situ FTIR spectrometry to investigate the response of such sensors to CO and N_2_ exposure by comparing sensors with and without exposure to CO [29]. According to some research, PANI blends with other non-conducting polymers can lead to some improvements in the mechanical, thermal, and in some cases electrical properties [32]. After optimizing the type of dopant, the dopant concentration, the PI content, and the temperature of the sensors, they have an improvement of the electrical sensitivity and a strengthening of the fragility of the PANI layer due to the introduction of PI. Interestingly, CO was not observed to form a chemical bond with PANI in this experiment. The proposed mechanism consists of a stable ^+^C≡O^−^ interaction between CO and PANI, where the negative charges of the oxygen atoms replace the negatively charged counter ions A^−^ at the positive polaron amine forms. Thus, the ^+^C≡O^−^ has the capacity to remove the lone pair electron at the amine nitrogen, leading to the formation of neutral carbon and the positive charging of amine nitrogen. Hence, electrical conductivity increases because of the increase in charge carriers within the polymeric structure. A diagram of this mechanism is presented in Figure 4.

**Figure 5:**
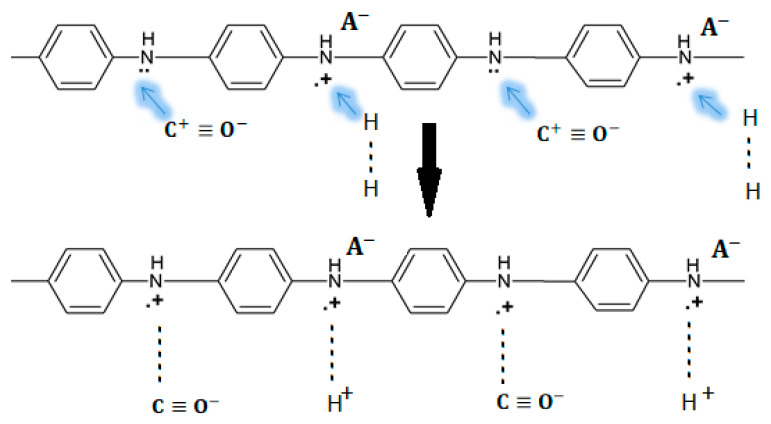
The mechanism proposed for interactions of CO and H_2_ with PANI [28].

Sensing layers using PANI are not restricted to the monitoring of a single gas species, with Liu et al. proposing a system for the simultaneous detection of CO and hydrogen (H_2_) in fuel cells [28]. This mechanism is possible due to CO and H_2_ being able to separately react with the amine groups in PANI in a double reaction. Thus, as can be seen from Figure 5, H_2_ reacts with the protonated amine nitrogen atoms within the PANI chain, whereas CO react with the unprotonated ones. While such sensors can be used to prevent catalyst poisoning in fuel cells, their application space is limited by the interference of atmospheric gases leading to the perturbation of the resistive signals.

A further sensing mechanism is shown by the sensor proposed by Hejczyk et al., which employs a surface acoustic wave mechanism for the detection of CO [30]. In such structures, gas molecules are bound within the polymer layer, leading to an increase in mass and thus in a shift in the resonant peak of the sensor. While such sensors can reach low limits of detection, the implementation of such mechanisms is limited by the requirement of high operation temperatures, 35 °C in synthetic air. Moreover, the sensor is prone to interference from other gases, such as oxygen, nitrogen, and water vapors in the air.

Recent research has shifted from the use of simple PANI sensing layers for the detection of CO with the use of NCMs composites being employed to address some of the weaknesses of simple layers. Thus, using carbon structures in the functionalization of the sensing layer can address issues with the low sensing area, interference of other gases, and humidity effects that lower the obtainable sensitivities and specificities of simple CP sensors. Moreover, improvements in formulation strategies and deposition techniques have also led to improvements in the performance of the sensors and need to be addressed in further sections.

### 3.3. PANI–NCM Structure Sensing Mechanism on CO Exposure

**Figure 6:**
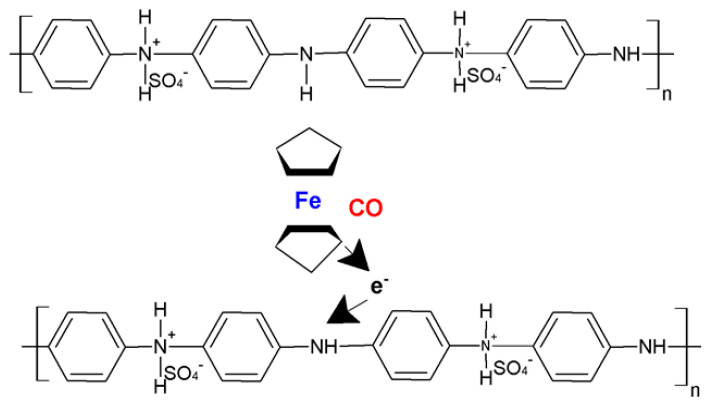
The sensing mechanism for PANI:PSS/SWCNT/Fc.

Current technologies functionalizing CPs with NCMs are restricted to the resistive measurements of changes in a target gas’s concentration. For polymeric structures that include insertions of CNTs, a drop in resistance is detected upon exposure to CO. A selection of sensors that employ either SWCNTs or MWCNTs for the detection of monoxide can be seen in Table 3, with the performance of each sensor being annotated in terms of range, sensitivity, response time, and operating temperature. While the increased area provided by the inclusion of CNTs leads to an increase in performance as opposed to polymers that simply undergo a doping process, the increase in sensitivity is dependent on the choice of deposition of the carbon nanostructures. Thus, deposition techniques such as drop casting and solvent casting lead to more sensitive sensors than the simple dispersion of the carbon elements in the polymer. For example, the PANI/CNT/PVA nanofibers (PVA, polyvinyl alcohol) developed by Wanna et al. display increases in sensitivity of up to two orders of magnitude due to added PVA [33]. The nanofibers were fabricated by electrospinning a MA-PANI/CNT compound with PVA, the result being then deposited on a series of aluminum (Al) interdigitated electrodes set on a glass substrate. Hence, it is important to analyze the effect of formulation strategies and deposition techniques on the performance of resistive chemosensors. Due to their high inherent sensitivity to hydrogen-containing gases, multifunctional sensors, for the detection of several species of gases, can be created if a sufficiently large sensing area is available. One such resistive sensor was proposed by Kim et al., who employed a SWCNT functionalized PANI layer for the simultaneous detection of NH_3_ and CO [34]. Such measurements are possible due to the different mechanisms of sensing employed in the detection of the two gases. While for CO, a resistive decrease is generated by the exchange of electrons between the PANI layer and the gas, for ammonia, an increase in resistance is generated via a reversible doping–undoping process. The sensor has been shown to achieve a limit of detection for CO of 5 ppm in gas mixtures with hydrogen gases, thus highlighting the importance of sensing mechanism in the selective detection of gases. Roy et al. make use of MWCNTs to obtain faster response times and higher absorption areas for the detection of CO with their PANI/MWCNT resistive sensor [31]. Measured CO is quantified using the Langmuir adsorption model, with the model being employed both for the calculation of the response to gas exposure and the identification of the recovery time of the sensor. Such models assume that only the top layer participates in the gas adsorption and CO molecules are adsorbed by the nitrogen atoms from the amide group of PANI [31]. Due to this physisorption, a charge transfer occurs in the PANI-MWCNT composite, leading to a decrease in the resistance of the sensor. The added MWCNT increases the surface area of the reaction with the CO molecules; however, the complexity of the carbon structure hinders the sensitivity of the structure when exposed to low concentrations of the target gas, making such sensors usable only in high-concentration environments of 500–1000 ppm. The detection of low concentrations of CO (<300 ppm) was enabled by Savin et al. by adding ferrocene (Fc) as a mediator to their PANI/SWCNT substrate [35]. Such structures have been observed to come with the additional benefit of limiting the effect of humidity on the sensor’s response. For this type of sensor, Fc mediates the electron transfer between the CO molecules and PANI, with the electrocatalytic properties of the sensor being enhanced by the strong π–π interaction between Fc and the SWCNTs [36]. The diagram of the mechanism of interaction between PANI, Fc, and CO molecules is presented in Figure 6.

### 3.4. PPy Structure and Conductivity

**Figure 7:**
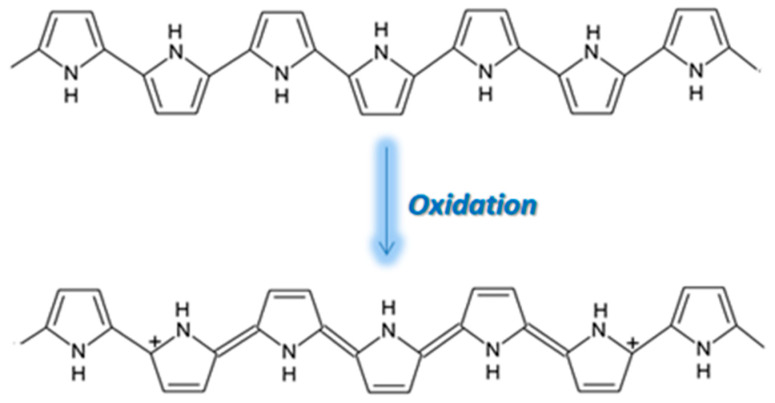
The oxidation of PPy [41].

PPy is an attractive alternative to PANI as a CP for gas sensing, displaying excellent sensitivity to gas molecules, tunable conductivity, a low production cost, and the ability to function at room temperature [37]. However, the use of PPy is not widespread due to its poor stability, the oxidation of PPy being highlighted in Figure 7. During oxidation, the removal of π electrons in the conjugated bond leads to a local relaxation of the benzenoid structure into a quinoid structure, thus creating a pair of radicals. This, together with the appearance of a positive charge, leads to the formation of bipolarons, leaving only two cations in the PPy ring. Those cations can move through the π electronic cloud, thus generating the conduction of electron through the sensing layers. Thus, unlike PEDOT and PANI, when PPy reacts with gases that possess electron acceptors, the electrons from its aromatic ring are removed and the conduction is enhanced [38,39,40].

#### 3.4.1. PPy Sensing Mechanism on CO Exposure

**Figure 8:**
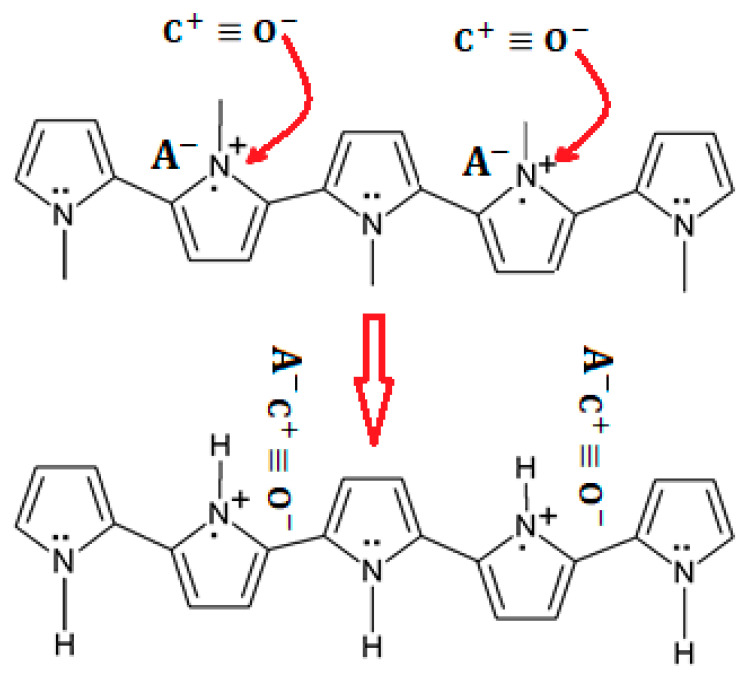
Scheme representing a possible sensing mechanism of the PPy-based sensor [42].

When a gas possesses electrons donors, such as in the case of CO, PPy takes electrons from the gas, leading to a decrease in the electrical resistance [38,39,40]. The reaction process of PPY with CO is shown in Figure 8 [42].

Table 4 presents the sensing performance of PPy on CO exposure. Compared to other CPs, there are few studies where PPy is employed for CO detection. Moreover, due to its poor stability, it is recommended to only use PPy as a component in composite materials.

**Figure 9:**
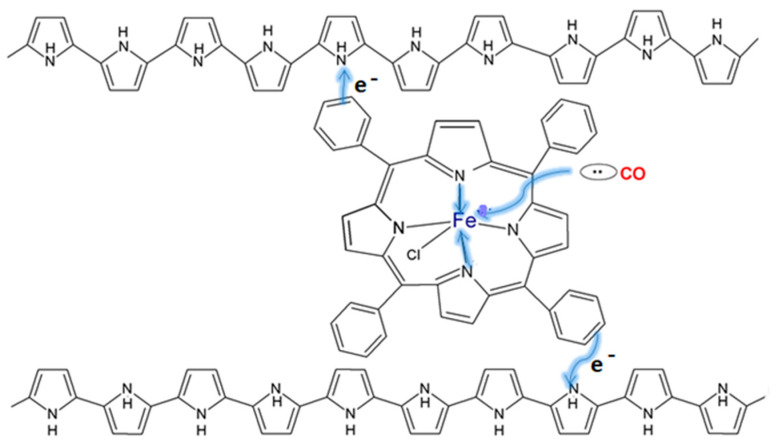
The scheme of binding CO with PPy-FeTPPCl [45].

Moreover, when used without carbon structures, PPy displays a drop in sensitivity when the operating temperature decreases, with an optimal operating temperature at which the PPy is stable being determined to be 300 °C [43]. One such sensor developed by Lee et al. for the dual gas detection of CO and NH_3_ uses a combination of acid-doped PPy and PSS to address the stability issue. While the sensor was validated for concentrations of CO and NH_3_ as low as 9 ppm, the system is proposed to preserve its performance even for sub-ppm limits of detection [44]. Thus, to preserve stability and show enhanced sensitivity and selectivity for CO detection, it is necessary for a PPy layer to be doped with dopants such as ferrocene derivates or to be chemically functionalized with porphyrin iron chloride derivates. Another such example is given by Santhosh et al., where they employed a PPy layer that was chemically functionalized with 1 mol% of 5,10,15,20-tetraphenyl-21H,23H-porphyrin iron chloride (FeTPPCl) for the detection of CO in the ppm level. Such CO sensing mechanisms are anchored around three reaction steps for the generation of a fast response signal: CO molecules interact very fast with iron metals from the center of the porphyrin complex, the Fe (III) reduction to Fe (II), and the conjugated system transferal of electrons into the PPy chains. The diagram of this mechanism is presented in Figure 9 [45].

**Figure 10:**
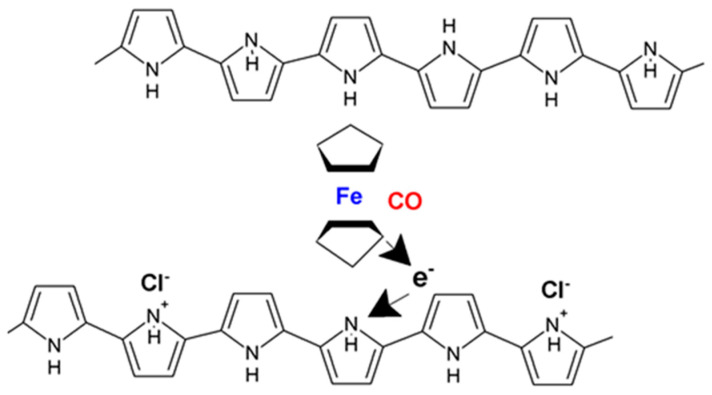
Interaction of CO with Fc and PPy [47].

Radhakrishnan and Santhosh also make use of a Fc-modified PPy layer to increase CO detection performance, with the Fc being set into the PPy layer by direct incorporation during polymerization [46]. A primary mechanism involves the formation of polarons through the interaction of the lone free electron of the nitrogen and the electron-withdrawing nature of the CO molecule. This combined with the formation of a ferrocenium ion donor–acceptor complex with FeCl_3_, due to the incorporation of Fc into PPy in the presence of FeCl_3_, led to an enhancement of the resistance signal. The scheme of this mechanism is presented in Figure 10.

Another rapid CO detection system was developed by Radhakrishnan et al. using a PPy layer chemically modified with ferrocenylmethyltrimethylammonium iodide, which achieved limits of detection of 300 ppm within 1 s of exposure to CO [47]. While the sensors using PPy and Fc-doped PPy show both excellent CO sensitivity and response time, further studies are necessary to assess the stability of such sensing layers. Thus, carbon structure functionalized PPy remains the main technique for employing PPy in gas sensing.

#### 3.4.2. PPy-NCM Structure Sensing Mechanism on CO Exposure

Both MWCNTs and SWCNTs have not been shown to function well for the detection of CO when used in combination with PPy due to their inherently high sensitivity to NH_3_. Such sensors would experience high increases in resistance on exposure to NH_3_, due to it being an electronic donating gas and the sensing system behaving as a p-type semiconductor. However, this is not true when the carbon structure of the NCMs is graphene oxide, with the performance of sensors using this combination being shown in Table 5.

Graphene-based polymer composites have been shown to display both higher mechanical properties and higher electrical properties when compared with other carbon-based polymer composites while at the same time not biasing them toward a subset of gases [42,48]. In one such study, Naikoo et al. developed a sensing layer consisting of hybrid zeolite X/rGO in combination with PPy for the sensing of CO [48]. Zeolite insertion was responsible for both a decrease in PPy degradation and an increase in the sensitivity to CO, while the reduced graphene was the main factor in increasing the performance of the sensor [42]. Zeolite content has also been determined to affect the response of the sensor to CO, with increases of 34.61% to 65.34% being recorded when taking measurements at 100 ppm CO. The sensing mechanism on CO exposure in the case of PPy combined with graphene oxide (GO) is based on replacement of the anions at the lone pair of the nitrogen of pyrrole –N•⁺H– [42]. The interaction between CO and the lone pair on the nitrogen would lead to the formation of a polaron and thus increase in resistance. The mechanism has the same diagram as that presented in this review in Figure 8.

### 3.5. PEDOT-Sensing Mechanism on CO Exposure

**Figure 11:**
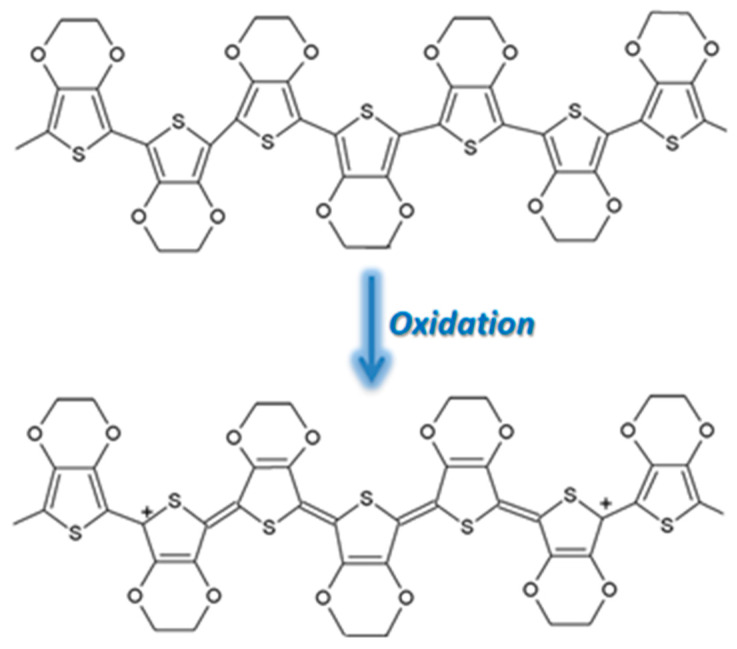
The oxidation state of PEDOT.

PEDOT is a very stable polymer, and in its oxidated state, shown in Figure 11, displays a high conductivity. Typically employed in combination with PSS, the compound is also soluble in water. The two cations of the oxidized PEDOT combine with the negatively charged PSS anions to yield a compound polymer that has high conductivity (10 $S\cdot {\mathrm{cm}}^{-1}$), transparency to visible light, and great stability [49].

**Figure 12:**
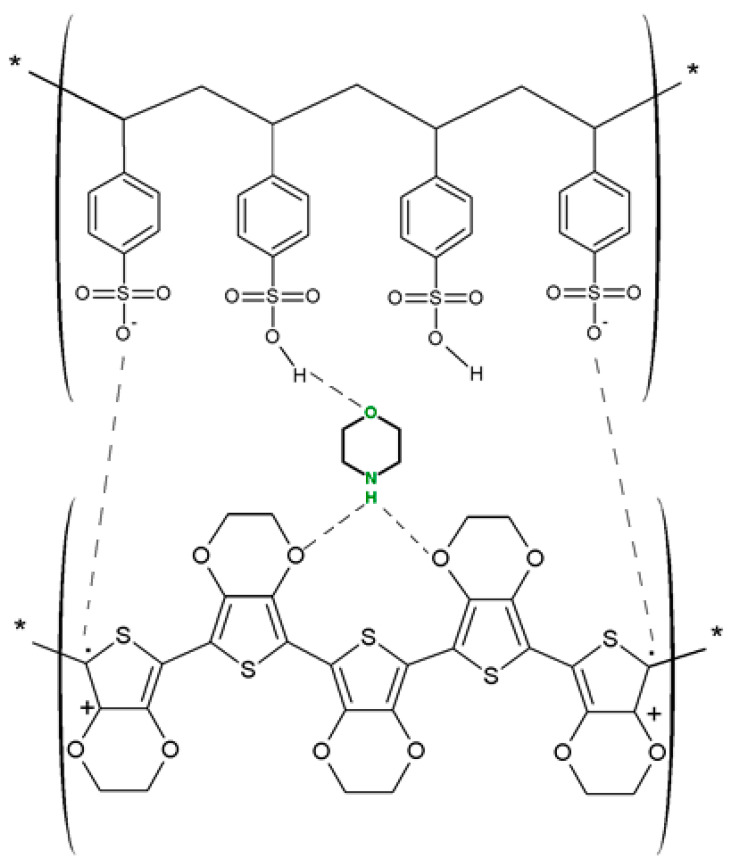
The schematic of a possible interaction of morpholine with PEDOT:PSS [49].

Most studies on the use of PEDOT:PSS as a gas detection layer focus on either the detection of NO_2_ or ammonia [50,51,52] due to the ease of oxidizing PEDOT with those gases. NO_2_ in particular is an oxidizing gas, that, when in contact with the π electron of polymers, results in the transfer of electrons from the polymer to the gas. However, PEDOT:PSS is very sensitive to changes in humidity with the studies of interest focusing on the removal of the interference of both perturbing gases and water vapor. Javadpour et al. proposed the creation of a CO sensor by adding Fe, Al, and morpholine to the polymer solution before the deposition process [49]. Morpholine three has shown to provide some robustness to interference, with stable morpholine forming a bridge between PEDOT and PSS, as shown in Figure 12.

**Figure 13:**
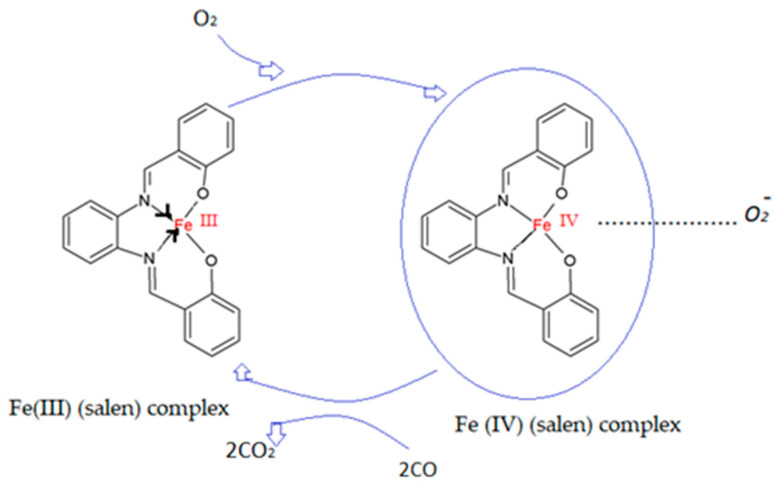
The possible mechanism for CO detection with PEDOT:PSS/Fe(salen) [55].

Thus, the bonding of stable morpholine provides robustness to interference from water vapors, as water molecules compete for the same bonding site. While doping the PEDOT:PSS polymer with Fe-Al-morpholine showed both an increase in CO selectivity and reaction time when compared to other compounds such as PANI with Fe-Al (10 s) [25] or PPy with Fc (100 s), the sensitivity to CO was not measured. A similar sensing layer, PEDOT:PSS with polyvinylpirolidone (PVP), was used by Hong-Di-Zhang et al. for the development of a quartz crystal microbalance for the detection of CO. The proposed system, while reaching low limits of detection, saturates more quickly when compared to chemosensors, despite using PVP to improve the contact area. The addition of PVP to the sensing layer in chemosensors leads to the sensitization of the layer to the interference of chemical vapors of ethanol, methanol, and acetone, thus reducing the specificity of the sensor to CO detection, when using PEDOT:PSS/PVP nanofibers for such a purpose [53]. Thus, increases in the sensing area, the addition of organic cycling molecules in the polymer mixture, and the addition of CO-affinity molecules are all techniques for increasing the sensitivity of a PEDOT sensor to CO. Table 5 presents the sensing performance of a number of PEDOT sensors on CO exposure. Memarzadeh et al. use cobalt (Co)-modified PEDOT:PSS as a layer for CO detection, the salen complex of Co being reported to improve the response and selectivity to CO [54]. Fe(salen)-based complexes were employed in a further two studies for improving the selectivity of PEDOT:PSS to CO [55,56]. The first study, by Arballoo et al., reports a high CO response of 31.32 ± 0.88% at room temperature (RT) in dry air and a low response time (38 s), while preserving the reversibility of the reaction. When comparing the reversibility of this sensor with the Co(salen)-doped PEDOT:PSS sensors, they concluded that their Fe(salen) showed an increase in reversibility, with less relative deviation being observed throughout the cycles. The desorption rate was also improved by using Fe(salen)-doped PEDOT:PSS. Typically, iron complexes have low tendencies to react with Co; however, they are prone to react with oxygen. So, for the system to react with CO, FeIII(salen) passes to FeIV (salen) in the presence of O_2_, with CO reaching a stable state CO_2_ due the interaction with the dioxygen Fe complex. Then, the oxygen that interacts with CO will be replaced by O_2_ from air, and reactions will continue repeating following the same reaction cycle. A diagram of this mechanism is present in Figure 13. The second instance of using a Fe(salen) system was employed by the same group in a study using Least Square Support Vector Machines (LSSVMs) to predict the response characteristics of the FeIII(salen)PEDOT:PSS under different conditions. The modeling results showed satisfactory agreements with the experimental results [57]. The performances of those sensors are shown in Table 6.

#### PEDOT-NCMs Structure Sensing Mechanism on CO Exposure

While two-dimensional polymer layers such as those of PEDOT:PSS have been shown to be selective to the detection of CO, the low density of adsorbed gas molecules leads to poor sensing responses [57]. Thus, by moving into the 3D domain through the addition of carbon structures can lead to both an increase in sensing area and an improvement in the charge-carrier transport between the gas molecules and the polymer, as holes in the conductive layer, a p-type semiconductor can be occupied by electrons donated by the CO molecules. One such sensing layer was proposed by Hyojae et al., where the introduction of MWCNTs resulted in an increased performance in the CO detection [58]. Thus, the PEDOT:PSS:MWCNT layer achieved detection ranges of 250–1000 ppm at room temperature, with results being comparable with those shown in previous sections [31].

## 4. Formulation Strategies

The use of CPs, with and without carbon materials, has two disadvantages: poor solubility in common solvents and mechanical instability, especially for PANI, due to changes during the oxidation and reduction reactions occurring during the doping process. One approach to improving solubility is to combine CPs with various hydrophilic polymers (or polyelectrolytes) such as PSS, PAA, PVP, and PEG, resulting in composite solutions such as PANI:PSS, PANI:PEG, PANI:PVP, PEDOT:PSS, and PPy:PSS [59,60,61,62,63,64].

Phongphut et al. presented the role of PSS, a co-solvent and dopant polyelectrolyte, in making a nanocomposite inkjet solution with PEDOT:PSS on carbon electrodes, with the resulting compound showing improved solubility of the polymer in aqueous media [65]. These electrolytes were also used for NCMs dispersions. For example, PSS was employed as a non-covalent method of carbon nanotube functionalization, with functionalization being followed by the polymerization with 3,4-ethylenedioxythiophene (EDOT). The role of PSS was not only to solubilize and disperse CNTs in aqueous solutions but also to bind the EDOT monomer to the CNTs surface, facilitating the achievement of a uniform coating with PEDOT. In their study, Biswas et al. used PSS as a supporting polyelectrolyte during the synthesis of various conjugated polymers, finding improvements in their processability and electrical transport properties [66]. PANI doped with PSS has received considerable attention due to its advantages in terms of easy synthesis, low cost, good thermal stability, and adequate electrical conductivity. Improving the mechanical stability of PANI and the PANI:PSS composite solution can be achieved by combining it with other carbon nanomaterials such as NCM (SWCNT/MWCNT) or graphene. NCMs has new properties such as good electrical conductivity, high electrical load-carrying capacity, and high chemical stability. In general, PANI has a stratified structure, but with the incorporation of carbon nanotubes, an interconnected mesoporous network structure is formed. This conductive nature of the PANI/carbon nanotube composite can increase the rate of transport of electrical charge carriers. As a result, the number of active sites will increase, which effectively increases the intra- and interlink charge mobility in the presence of electron donor or acceptor gases. The challenge of using carbon materials remains the obtaining of a good dispersibility of NCMs. For example, for SWCNTs, a series of hydrophobic carbon structures insoluble in most solvents, this dispersibility problem can be solved by finding dispersion enhancement methods such as SWCNT/MWCNT surface functionalization, the addition of surfactants (S27000 for Inkjet printing ink), ultrasonication, and association with other polymers, biomolecules, and organic acids. Ionic surfactants such as SDS and SDSB are also used for inhibiting the tendency of CNTs to aggregate in water [67,68,69]. Nonionic surfactants such as Triton X-100, Tween-80, Tween-60, and Tween-20 can also be used, but they lack Coloumb repulsion to prevent the aggregation of CNTs. The presence of long PEG chains increases the dispersion efficiency in solution with their molecular weight, with surfactant molecules entering the spaces between the tubes, preventing their regrouping. The more hydrophobic the surfactants, the less they prevent the aggregation of CNTs. Graphene dispersion is also dependent on the exfoliation procedure, and when obtained by exfoliating the liquid phase of graphite in water, the graphene obtained can be dispersed with the following surfactants: SDS, SDBS, LDS, cetyltrimethyl ammoniumbromide (CTAB), TTAB, SC, sodium deoxycholate (DOC), sodium taurodeoxycholate (TDOC), IGEPAL CO-890, Triton X-100, Tween 20, and Tween 80 [70,71].

## 5. Conclusions and Future Perspectives

In this review, an analysis on the performances and sensing mechanisms of CO detectors based on CPs and carbon composites has been conducted. Most of the sensors analyzed in the review showed CO absorption sites in their structure, with the Langmuire adsorption model offering a good description of their reaction mechanism. While sensors using NCMs based on metal oxides showed a non-uniformity in their resistive change to the presence of CO, with either an increase or decrease being possible, CP-based sensing layers have been found to consistently be characterized by a decrease in resistance. This consistency is due to the physical expansion of the polymer layer as well as due to the enhancement of the electron transfer properties resulting from the functionalization with CNTs. Thus, the advantages of using CPs have been determined to include higher selectivity, shorter response and recovery times, and operation at room temperature. However, research still needs to be conducted on the interferences of other gases and the effect of humidity. Based on the presented data, we can conclude that hybrid materials formed by conductive organic polymers combined with carbon nanostructures are desirable materials for use in gas detection, especially in CO detection. Although chemoresistive sensors have been extensively studied in recent decades, there are some aspects that can be improved: methods of preparation and deposition of the sensitive film, both in terms of cost and technology, sensitivity to non-intrinsic gas species, selectivity to a chosen gas, and the degree of miniaturization of sensors and their control devices.

## Figures and Tables

**Table 1:** Name and chemical structure of CPs and nanostructures carbon materials (NCMs).

Name of CP and NCMs	Abbreviation	Chemical Structures	
Polyaniline	PANI	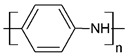 (a)	
	PANI:PSS/SWCNTs	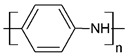	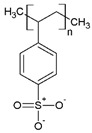
		(a)	(b)
		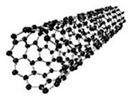 (c)	
	PANI:PSS/MWCNTs	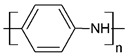	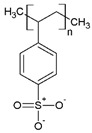
		(a)	(b)
		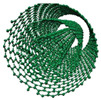 (d)	
	PANI:PSS/SWCNT/Fc	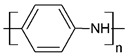	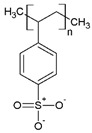
		(a)	(b)
		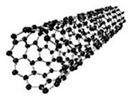	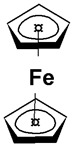
		(c)	(e)
	PANI/rGO	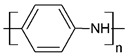	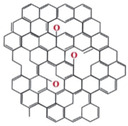
		(a)	(f)
Poly(3,4-ethylenedioxy)thiophene	PEDOT	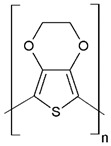 (g)	
	PEDOT:PEG/SWCNTs	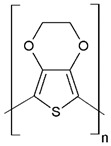	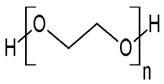
		(g)	(h)
		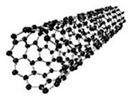	
		(c)	
	PEDOT:PSS/MWCNTs	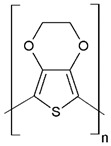	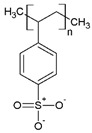
		(g)	(b)
		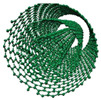	
		(d)	
	PEDOT/rGO	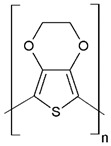	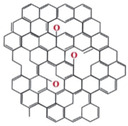
		(g)	(f)
Polydiphenylamine	PDPA/MWCNTs	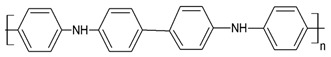 (i) 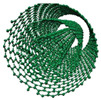 (d)	
Poly(dyallyldimethyl-ammonium chloride)	PDDA/MWCNTs	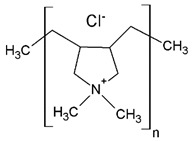	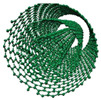
		(j)	(d)
Polypyrrole	PPy	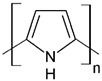 (k)	
	PPy/GO or rGO	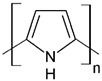	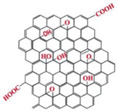
		(k)	(l)
		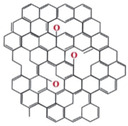	
		(f)	

(a) polyaniline (PANI), (b) sodium polystyrene sulfonate (PSS), (c) single-walled carbon nanotubes (SWCNTs), (d) multi-walled carbon nanotubes (MWCNTs), (e) ferrocene (Fc), (f) reduced graphene oxide (rGO), (g) poly(3,4-ethylenedioxy)thiophene (PEDOT), (h) polyethylene glycol (PEG), (i) polydiphenylamine (PDPA), (j) poly(dyallyldimethyl-ammonium chloride) (PDDA), (k) polypyrrole (PPy), (l) graphene oxide (GO).

**Table 2:** Overview of the PANI-based sensors for CO detection.

Sensing Materials	Doping Agents	Concentration Range (ppm)	Response Time	Sensitivity	Operating Temp (°C)	Response Formula	References
Nanocrystalline PANI	HCl	0.02–30 ppm	8–10 s	400–600	RT	*S* * = Ie/Io,	[25]
PANI	HCl/Fe and Al	0–150 ppm	5 s	800	RT	*S* = (Ie − Io)/Io	[26]
PANI	Maleic acid	100–500 ppm	1.1 min	0.01—0.03	RT	$S=\frac{\left|\Delta R_{g}\right|\;}{R_{g}}$	[27]
PANI	CSA **	1–100 ppm	-	−24%	-	$\frac{\Delta R}{R_{0}}\left(\%\right)$	[28]
PANI horizontal nanofiber	HCl	1–100 ppm	-	−18% for 1ppm	-	$\frac{\Delta R}{R_{0}}\left(\%\right)$	[28]
PANI/PI	CSA HNO_3_	1–1000 ppm 1–1000 ppm	36.8 min for 1000 ppm	0.338 S·cm^−1^; 1.040 S·cm^−1^	25 °C–55 °C at 1 atm	$\frac{\Delta \sigma }{\sigma_{N_{2}}}$ ***	[29]
PANI/ Nafion	NA	0–20 ppm		5 ppm in synthetic air	34 °C; 42 °C	Δ*F* [Hz]	[30]

* = sensitivity, ** = camphorsulfonic acid, *** σ=conductivity (S·cm^−1^); *σ*_N2_ = the electrical conductivity value when exposed sensor to N_2_; Δσ = average values obtained from two to four different samples at a specific value of temperature.

**Table 3:** Overview of the NCMs with PANI and carbon nanomaterials used for CO detection.

Sensing Materials	Doping Agents	Concentration (ppm)	Response Time	Sensitivity	Operating Temp (°C)	Response Formula	References
PANI/PVA/fiber/CNTs	MA	100 ppm–500 ppm	NA	1.5–3.5	RT	$S=\frac{\left|R_{g}-R_{a}\right|\;}{R_{a}}$	[33]
PANI/dispersed CNTs	MA	100–1000 ppm	0.6 min	0.04–0.12	RT	$S=\frac{\left|R_{g}-R_{a}\right|\;}{R_{a}}$	[27]
PANI/SWCNTs	HCl	5 ppm; 80 ppm	NA	NA	RT	$S\left(\%\right)=\frac{\left|R_{g}-R_{0}\right|\;}{R_{0}}\times 100$	[34]
PANI/MWCNT	HCl	500–1000 ppm	76 s	6.8–26.7%	RT	$S=\frac{\left|R_{g}-R_{a}\right|\;}{R_{a}}$	[31]
PANI:PSS/SWCNT/Fc	H_2_SO_4_	0–300 ppm	33 s for >100 ppm 30 s for <100ppm	6–55%	RT	$S=\frac{\left|R_{g}-R_{a}\right|\;}{R_{a}}\times 100$	[35]

**Table 4:** Sensor’s performance based by the PPy layer.

Sensing Materials	Concentration Range (ppm)	Response Time	Sensitivity	Operating Temp (°C)	Response Formula	References
PPy	100–500 ppm	8–10 s	6.5% for 500 ppm	300 °C	$\frac{\Delta R}{R_{0}}\left(\%\right)$	[43]
PPy:PSS	9 ppm	-	≈1% for 9 ppm	RT	$\frac{\Delta R}{R_{0}}\left(\%\right)$	[44]
PPy-FeTPPCl	100–300 ppm	500 s	12% for 100 ppm	RT	$\frac{\Delta R}{R_{0}}\left(\%\right)$	[45]
PPy-Fc	300 ppm	t_50_ = 96s	25.8% for 300 ppm	RT	$\frac{\Delta R}{R_{0}}\left(\%\right)$	[46]
PPy-Fc derivates	300 ppm	t_50_ = 0.43 s	12% for 300 ppm	RT	$\frac{\Delta R}{R_{0}}\left(\%\right)$	[47]

**Table 5:** Sensors performance for CO detection using PPy-NCMs.

Sensing Materials	Concentration Range (ppm)	Response Time	Sensitivity	Operating Temp (°C)	Response Formula	References
Zeolite-X/rGO/PPy	5–1000	303 s–600 s	14.9–77.4%	RT	$\frac{\Delta R}{R_{0}}\left(\%\right)$	[48]
PPy/rGO	50–300	89s	45%	RT	$\frac{\Delta R}{R_{0}}\left(\%\right)$	[42]

**Table 6:** Sensors performance for CO detection using PEDOT:PSS.

Sensing Materials	Concentration Range (ppm)	Response Time	Sensitivity	Operating Temp (°C)	Response Formula	References
PEDOT:PSS/ Morpholine	-	5 s	-	Vacuum/mixing air and CO	Percent of resistance variation relative to the base (in vacuum) resistance of thin film	[49]
PEDOT:PSS/Co (salen)	-	-	-	RT	$\frac{\Delta R}{R_{0}}\left(\%\right)$	[54]
PEDOT:PSS/Fe (salen)	10–100 ppm	38 s	1.50	RT	$\frac{R_{air}}{R_{gas}}$	[55]

## Abbreviations

CO—carbon monoxide, CNTs—carbon nanotubes, PANI—polyaniline, PPy—polypyrrole, PEDOT—poly(3,4-ethylenedioxythiophene), PTh—polythiophene, SWCNTs—single-walled carbon nanotubes, MWCNTs—multi-walled carbon nanotubes, GO—graphene oxide, rGO—reduced graphene, CP—conductive polymers, NCMs—nanocomposite materials, PSS—polystyrene sulfonate, PAA—polyacrylic acid, PVP—polyvinylpyrrolidone, PEG—polyethylene glycol, CSA—camphorsulfonic acid, NA—Nafion, MA—maleic acid, PVA—polyvinyl alcohol, PI—polyimide.
